# Optimising the implementation of digital-supported interventions for the secondary prevention of heart disease: a systematic review using the RE-AIM planning and evaluation framework

**DOI:** 10.1186/s12913-023-10361-6

**Published:** 2023-12-04

**Authors:** Caroline de Moel-Mandel, Chris Lynch, Ayuba Issaka, Justin Braver, Georgios Zisis, Melinda J. Carrington, Brian Oldenburg

**Affiliations:** 1 School of Psychology & Public Health, La Trobe University, Melbourne, VIC Australia; 2 Baker Heart and Diabetes Institute, Melbourne, VIC Australia; 3 NHMRC CRE in Digital Technology to Transform Chronic Disease Outcomes, Melbourne, VIC Australia; 4 Northern Health, Melbourne, VIC Australia; 5 Baker Department of Cardiometabolic Health, Faculty of Medicine, Dentistry and Health Sciences, The University of Melbourne, Melbourne, VIC Australia; 6 Western Health, Melbourne, VIC Australia

**Keywords:** Implementation evaluation, Systematic review, Health care, eHealth, mHealth, Secondary prevention, RE-AIM

## Abstract

**Background:**

mHealth technologies are now widely utilised to support the delivery of secondary prevention programs in heart disease. Interventions with mHealth included have shown a similar efficacy and safety to conventional programs with improvements in access and adherence. However, questions remain regarding the successful wider implementation of digital-supported programs. By applying the Reach-Effectiveness-Adoption-Implementation-Maintenance (RE-AIM) framework to a systematic review and meta-analysis, this review aims to evaluate the extent to which these programs report on RE-AIM dimensions and associated indicators.

**Methods:**

This review extends our previous systematic review and meta-analysis that investigated the effectiveness of digital-supported programs for patients with coronary artery disease. Citation searches were performed on the 27 studies of the systematic review to identify linked publications that reported data for RE-AIM dimensions. All included studies and, where relevant, any additional publications, were coded using an adapted RE-AIM extraction tool. Discrepant codes were discussed amongst reviewers to gain consensus. Data were analysed to assess reporting on indicators related to each of the RE-AIM dimensions, and average overall reporting rates for each dimension were calculated.

**Results:**

Searches found an additional nine publications. Across 36 publications that were linked to the 27 studies, 24 (89%) of the studies were interventions solely delivered at home. The average reporting rates for RE-AIM dimensions were highest for effectiveness (75%) and reach (67%), followed by adoption (54%), implementation (36%) and maintenance (11%). Eleven (46%) studies did not describe relevant characteristics of their participants or of staff involved in the intervention; most studies did not describe unanticipated consequences of the intervention; the ongoing cost of intervention implementation and maintenance; information on intervention fidelity; long-term follow-up outcomes, or program adaptation in other settings.

**Conclusions:**

Through the application of the RE-AIM framework to a systematic review we found most studies failed to report on key indicators. Failing to report these indicators inhibits the ability to address the enablers and barriers required to achieve optimal intervention implementation in wider settings and populations. Future studies should consider alternative hybrid trial designs to enable reporting of implementation indicators to improve the translation of research evidence into routine practice, with special consideration given to the long-term sustainability of program effects as well as corresponding ongoing costs.

**Registration:**

PROSPERO—CRD42022343030.

**Supplementary Information:**

The online version contains supplementary material available at 10.1186/s12913-023-10361-6.

## Background

Cardiac rehabilitation (CR) is a multi-component program that is designed to optimise cardiovascular risk reduction, foster compliance to healthy behaviours, and promote an active lifestyle for people with cardiovascular disease [1, 2]. Introduced in the late 1960s, there is a substantive evidence base supporting CR as a clinically effective and cost-effective intervention for the secondary prevention of heart disease [3, 4] and it is now routinely recommended across a wide range of cardiac diagnoses. While centre-based CR programs have been shown to be effective in reducing hospital admissions and improving health-related quality of life [3, 5], reported referral, access, and participation rates have been sub-optimal [6, 7].

The use of mobile health (mHealth) technologies in support of CR and secondary prevention programs has increased in recent years. An increase accelerated by the onset of the COVID-19 pandemic when access to centre-based programs was limited. Integrating mHealth, which includes mobile and wireless technologies, wearables, mobile apps [8], and more recently, the use of sensors and AI [9], into home-based and hybrid models of secondary prevention has shown similar safety and efficacy to centre-based programs [10]. Such digital-supported secondary prevention programs have shown increases in adherence [11] and positive effects on behavioural and psychosocial outcomes [11, 12]. However, unifying evidence for the overall impact of these programs on health outcomes is lacking, especially in programs that incorporate novel technologies [11].

A recent systematic review and meta-analysis examined the impact of digital-supported secondary prevention programs, assessing if programs using mHealth reduced readmissions and mortality in patients with coronary artery disease [13]. The review found that programs using mHealth may be effective in lowering hospital visits and readmissions, but there was no evidence for reduced mortality outcomes. The authors reflected that assessment of longer-term effectiveness and program scalability may be hindered by non-generalisable study populations and short follow-up periods. Thus, while digital supported secondary prevention programs have become more common and have demonstrated efficacy, translatability beyond the research setting is unclear.

The Reach-Effectiveness-Adoption-Implementation-Maintenance (RE-AIM) model has proven to be a useful planning an evaluation framework to evaluate the degree to which interventions report on internal (i.e., accuracy of research methods and findings) as well as external validity (i.e., generalisability and translatability) factors [14]. RE-AIM has been successfully applied to systematic and scoping reviews that have evaluated threats to the transferability of a multitude of digital-supported health interventions to practice, including those for mental health [15, 16], diabetes self-management [17], chronic disease [18], physical activity [19], and vaccination promotion [20]. To our knowledge, there has been no review reporting on the implementation of digital-supported interventions to improve health outcomes and the secondary prevention of heart disease.

This study aims to evaluate the extent to which RE-AIM dimensions and associated internal and external validity indicators are reported in the included studies of the systematic review and meta-analysis by Braver et al. [13], as such knowledge would offer guidance to improve the wider implementation and optimisation of digital-enabled secondary prevention programs.

## Methods

### Study design

We undertook a systematic search for any additional publications to those already included in the systematic review and meta-analysis by Braver et al. [13]. Braver included studies that compared mHealth-supported secondary prevention programs against standard delivered programs, this study searched for and included any additional publications that reported on characteristics, delivery, and implementation of those studies.

This study is registered with PROSPERO (CRD42022343030) and conforms to the Preferred Reporting Items for Systematic Reviews and Meta-Analyses (PRISMA) guidelines [21].

### Search strategy and selection

To identify additional publications to those already included in the systematic review and meta-analysis by Braver et al. [13], we conducted reference list and citation searches using the MEDLINE, Embase, and Scopus databases. We also searched trial registries using identified clinical trial registration numbers. All search results were imported into Endnote 20 software and duplicate records were removed. Remaining records were exported into Covidence, a cloud-based systematic review program [22].

Titles and abstracts were examined for eligibility independently by two reviewers (AI and CL), with a third reviewer (CM) adjudicating any discrepancy. Non-English language publications and publications reporting on work other than the original studies included by Braver et al. were excluded. Conference abstracts, reviews, book chapters were also excluded. The full text of the remaining publications was obtained and screened. Publications were included if they described any additional data to that reported in the studies included studies of Braver et al., such as secondary analyses, long-term follow-up results, or cost-effectiveness analyses. The inclusion criteria for eligible studies are presented in Table 1.

**Table 1:** Inclusion criteria of the systematic review

Inclusion criteria	Description
Language	English, published in a peer-reviewed journal
Study design	Randomised controlled trials or prospective cohort studies
Sample size	Minimum 50 participants
Follow-up	At least 30-days follow-up
Participants	Patients discharged from hospital with CAD (not heart failure)
Intervention	Disease management program using mHealth, focusing on more than one behaviour
Control/comparison group	Usual care
Outcome	All-cause or cardiovascular mortality, all-cause or cardiovascular readmissions, or major adverse cardiovascular events

Braver et al. initially identified 27 unique studies to be included in their systematic review, they later decided to exclude nine studies as there was no usual care disease management plan as the control group, however, we included all the 27 unique studies in our review since the control group program was not relevant to our study aim. The full search strategy of Braver et al. is described elsewhere [13].

### Data extraction tool

The RE-AIM data extraction tool used was adapted from previous extraction tools [17, 19] to suit the characteristics of digital-supported interventions and align with the recommendations made by Holtrop et al. [23]. Minor modifications were made to previously used RE-AIM item definitions to ensure that the description of the indicators for each of the five RE-AIM dimensions were relevant and unambiguous [16, 19]. The resulting 25-item coding sheet, consisting of the five RE-AIM dimensions and corresponding indicators is presented (Table 2).

**Table 2:** Adapted RE-AIM framework dimensions with internal and external validity indicators

**Reach—Individual level**	**The number, proportion, and representativeness of participants**
Target population	The process by which the target population was identified and recruited for participation in the intervention
Inclusion criteria	Characteristics that determined if a potential participant was eligible to participate
Exclusion criteria	Characteristics that determined if a potential participant was not eligible to participate
Participation rate	Sample size divided by the number of eligible participants exposed to recruitment strategies
Reasons for not participating	Reasons provided for not participating in intervention
Representativeness	Comparison of characteristics of the study participants to target population
**Effectiveness—Individual level**	**The impact of the intervention on important individual outcomes, including quality of life, negative outcomes and on attrition**
Primary outcome of intervention	Description of primary outcome measure
Secondary outcome of intervention	Description of secondary outcome measure
Quality-of-life as secondary outcome	The study includes a measure of the quality of life as secondary outcome measure
Results for at *least* one follow-up	The study reports on study variable(s) measured at specific time point(s) after baseline measures
Intent-to-treat analysis utilised	The study reports it analyses all participants, regardless of whether they received or adhered to the allocated intervention, or if it only includes participants that were present at follow-up
Satisfaction with intervention	The study includes a patient satisfaction survey or monitors patients’ feedback
Negative outcomes	Negative outcomes are assessed to evaluate unanticipated consequences that may be a direct product of the intervention (barriers to participate)
Percent attrition	The proportion that was lost to follow-up or dropped out of the intervention (including deaths)
	Provided reasons for dropping out of the intervention
**Adoption—Settings and staff levels**	**Setting and staff factors that relate to the adoption of the intervention**
Description of intervention location	The characteristics of the location of the actual intervention (home, centre-based, hybrid, or other)
Staff required to deliver the intervention	Did the intervention require staff to deliver (parts of) the intervention?
Further details of staff providing intervention (if applicable)	Characteristics of these intervention delivering staff members Level of expertise of these intervention delivering staff members Uptake/Adoption rate of staff
**Implementation—Settings and staff levels**	**The degree to which the intervention is delivered as intended time and cost of delivery and implementation strategies**
Intervention duration and frequency	Length the intervention
	Frequency: number of contacts with participants
Fidelity	Was the intervention delivered as intended or amended post protocol (consistency of delivery)
	Details provided of amended protocol if applicable (type, timing, and reasons)
Measures of cost of implementation	The ongoing cost (e.g., money, time) of the implementation across all levels of the intervention
Theory-based approach	Was the implementation informed by theory? Name of theory used if applicable
**Maintenance—Individual and setting levels**	**The degree to which the intervention is maintained as well as the long-term effects of the program on outcomes**
Indicators of program maintenance	Description of program continuation after completion or reasons provided for program discontinuation. If program continued, were adaptations made post study?
Program adaptation in other settings	The intervention is adopted in other settings
Indicators of maintained behaviour	Report on outcome measures of individuals at some duration after intervention termination Description of assessed outcomes post-intervention
Measures of cost of maintenance	The ongoing cost of maintaining delivery across all levels of the intervention

Source: Adapted from Glasgow’19, Blackman’13, Yoshida’20 and Holtrop’21

For reach, the coding sheet included the target population, study inclusion and exclusion criteria, participation rate, and the representativeness of the participants. One indicator was added to assess reasons for not participating, as this information might provide insight into any unanticipated negative consequences of the intervention. Effectiveness was evaluated with eight indicators, including the impact of the intervention (primary and secondary outcomes, as well as measures of quality of life); the reporting of results for at least one follow-up; intent to treat assessment; intervention satisfaction; any unanticipated consequences; and participant attrition. Adoption consisted of three indicators. Two related to the intervention location (home, centre-based, hybrid, or other) and the staff delivering the intervention. One item was added to collect information about the individuals delivering the intervention, such as their characteristics, qualifications, level of expertise and uptake. The indicators used to assess implementation included duration and frequency of intervention, the ongoing cost of intervention delivery, and the extent to which the intervention was delivered as intended. We expanded this final item by asking for details of any adaptations made, to get insight into how the intervention worked in specific contexts [24]. In addition, we included an item to document whether theory-based approaches were used, as it is recognised that when a study is informed by theory, the probability of successful implementation and sustainability of an intervention increases [23, 25]. Finally, to document maintenance, four indicators were used, which included description and reasons for program (dis)continuation after completion, and measures of cost of maintenance. One item was added to indicate if the study reported on the maintenance of any behaviour change post-intervention [23]. Another item assessed if the intervention was adopted in a different setting [26].

### Data extraction

All included publications were coded independently in Covidence by two reviewers (AI and CM). Any discrepancy in the coding was resolved through discussion amongst the two reviewers and arbitration by a third reviewer (CL) until consensus was reached. We coded data that was related to general study and intervention characteristics and whether a study, including their associated papers, reported on each of the 25 RE-AIM indicators (i.e., yes or no). For each included study and their associated papers, we calculated an overall indicator reporting rate based on the total number of reported indicators (maximum score = 25). In addition, we calculated the indicator reporting rate for each RE-AIM dimension by dividing the total of “yes” scores for that dimension by the total number of indicators in the dimension. The reporting comprehensiveness of the studies was assessed using tertile cut-off points, as done in previous reviews using RE-AIM [27]. Studies that scored between 18–25 indicators (72–100%) were considered to have a high reporting quality, studies that scored 9–16 indicators (36–68%) were of moderate quality, and those that scored 0–8 indicators (0–31%) reflected low quality. In addition, we calculated the average reporting rates of the indicators for each dimension.

## Results

**Fig. 1:**
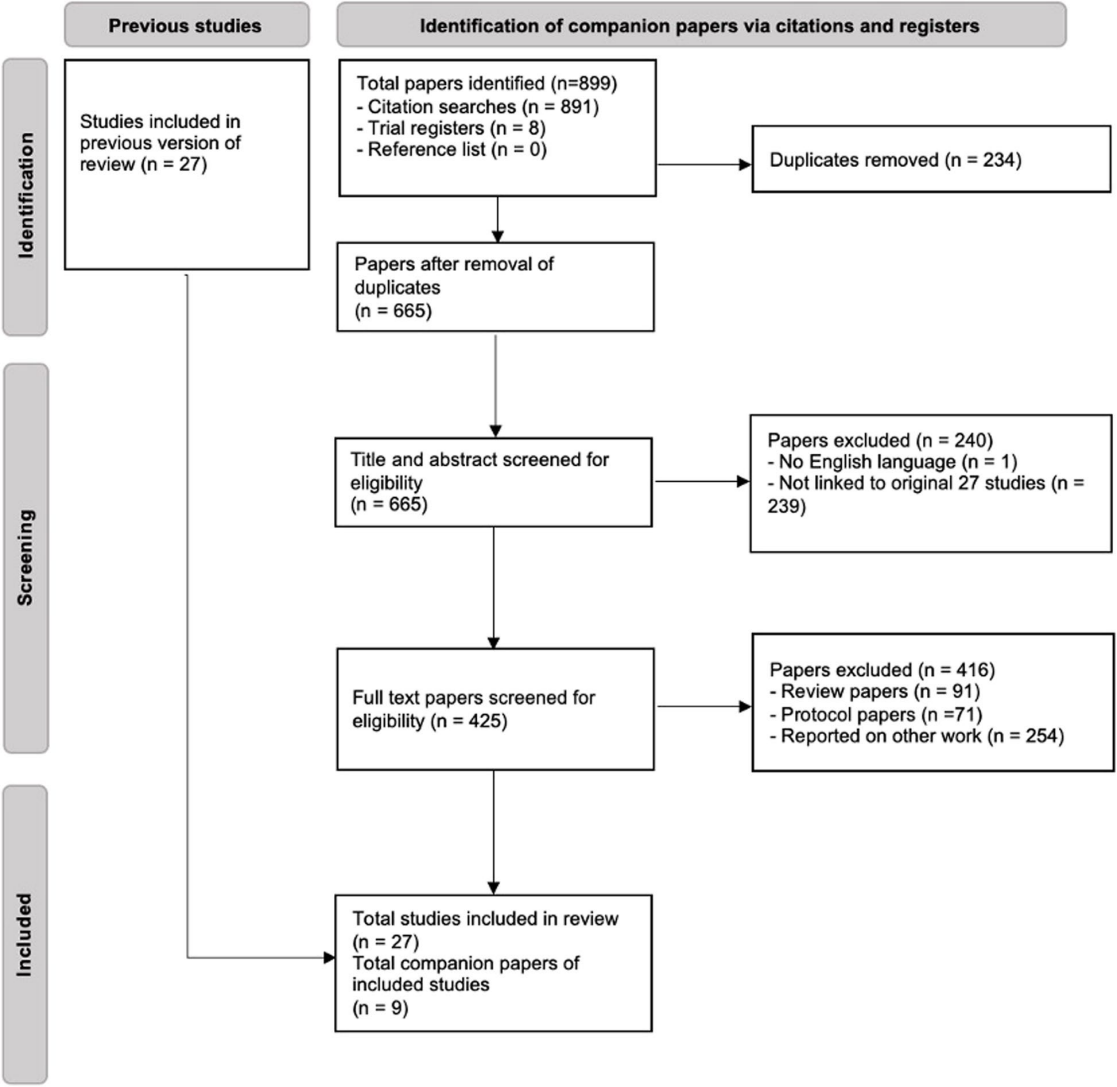
PRISMA flow diagram of the search strategy

Searches for additional publications to those already included in the systematic review and meta-analysis by Braver et al. [13], found 665 possible additional publications. Of these, nine were included in our study, resulting in a total of 36 publications linked directly to the 27 studies included in Braver et al. The nine additional publications provided data on one or more dimensions of the RE-AIM framework. An overview of the selection process is illustrated in Fig. 1.

### Study characteristics

Thirty six publications linked to the 27 studies were published between 2015 and 2022 (*n* = 30; 83%) and the earliest publication [28] was dated 2008. The characteristics and RE-AIM reporting rates extracted from the 36 publications of the 27 studies are presented in detail in an additional file (see Supplementary Table).

Of the 27 studies, most were conducted in Canada [28–32] and the USA [33–37] (*n* = 10; 41%) and Europe (*n* = 7; 26%), and all except one [34] (96%) were RCTs. Most study interventions (*n* = 24; 89%) were solely delivered in the home setting. Three studies used a hybrid delivery method, i.e., they were either delivered at a centre and included a home-based digital health component [37], or the intervention begun at the centre and continued at home [38, 39]. In 11 (46%) of the 24 solely at home delivered interventions [31–35, 40–45], no staff were directly involved in the delivery of care or they were only available on participant’s request. Seven studies [33, 35, 45–49] (26%) only used a mobile application to deliver the intervention, while three studies [37, 44, 50] (11%) used a mobile application and a web-based platform. Only four studies [28–30, 34] (15%) did not require the use of a smartphone during the intervention. In 10 studies (37%), the intervention included additional technology, including wearable devices [29, 33, 34, 38, 39, 43, 47, 50, 51] and electronically monitored pill bottles [36].

### RE-AIM reporting

The average reporting rates (% of RE-AIM dimension indicators) for the 27 studies were highest for Effectiveness (75%) and Reach (67%), followed by Adoption (54%), Implementation (36%) and Maintenance (11%) (see Table 3 and Additional file 1). In only one study [51], the comprehensiveness of reporting was considered high quality (72–100%). All other studies were rated as moderate quality (36–68%), although one study [34] was borderline moderate with a quality rating of 36%. The following sections summarise the reporting of the separate studies on each of the RE-AIM dimension indicators.

**Table 3:** Proportion of studies (*n* = 27) reporting on RE-AIM dimensions and elements

RE-AIM dimension and elements	Reporting rate (%)^a^
Reach (total)	67
Target population	0
Inclusion criteria	100
Exclusion criteria	93
Participation rate	89
Reasons for not participating	74
Representativeness	0
Effectiveness (total)	75
Primary outcome intervention	100
Secondary outcome intervention	96
Quality-of-life as secondary outcome	56
Results for at *least* one follow-up	52
Intent-to-treat analysis utilised	81
Satisfaction with intervention	63
Negative outcomes	0
Percent attrition	93
Adoption (total)	54^b^
Description of intervention location	100
Staff required to deliver the intervention	63
Further details of staff providing intervention (if applicable) (*n* = 18)^b^	0
Implementation (total)	36
Intervention duration and frequency	100
Fidelity	11
Measures of cost of implementation	0
Theory-based approach	33
Maintenance (total)	11
Indicators of program maintenance	7
Program adaptation in other settings	0
Indicators of maintained behaviour	37
Measures of cost of maintenance	0

^a^ Proportion is based on a denominator of 27 studies, reported across 36 articles

^b^ The reported percentage only relates to studies that required staff to deliver the intervention (*n* = 18). This influences the total reporting rate of the Adoption dimension

### Reach

Most studies (*n* = 24; 89%) indicated that they identified and recruited their participants during hospitalisation, shortly after discharge or in a rehabilitation centre. Lear et al. [29] did not provide details beside from evaluating participants from regional/rural settings, Volpp et al. [36] recruited participants via insurance companies’ data, and Wolf et al. [44] recruited from a previous study. No studies reported characteristics of target populations, as all potential participants were identified via disease characteristics. In some cases, further characteristics were provided in the inclusion and exclusion criteria, including age, language, and severe physical and/or mental impairments. All studies reported on both inclusion and exclusion criteria, however, only 11 studies (41%) provided information relevant to the use of mHealth, such as the requirement to own a (smart) phone [33, 40, 41, 45, 46, 52, 53], have a landline telephone or cellular service [31, 32], or have access to the Internet [30, 37]. One study [54] excluded participants (without a support person) that were physically or mentally unable to use the technical equipment needed for telemonitoring. Participation rate could not be calculated for three studies [28, 35, 55] as the authors did not include data on sample size or eligible participants exposed to the intervention. The median participation rate, from the 24 (89%) studies that reported participation rates was 41% (range, 6%—100%). None of the 27 studies comprehensively described all relevant characteristics of their participants, making it challenging to draw conclusions about the representativeness of the sample or whether the low participation rates indicated that the intervention was not suitable for the target population.

### Effectiveness

All except one study [44] reported on both primary and secondary outcome measures. Fourteen (52%) studies reported on study variable(s) for more than one follow-up time point [28–30, 35, 37–39, 43, 46–48, 50, 51, 55]. A little over half of the studies (*n* = 15; 56%) included the quality of life as an outcome measure [28, 30, 37, 39, 41, 42, 45–48, 50, 51, 54, 55] and 17 (63%) studies reported any measure of participant satisfaction or monitored participant feedback [28, 29, 31, 32, 34, 37, 39–43, 48, 49, 51, 53–55]. Maddison et al. [50] was the only study to report outcomes that might have been unanticipated consequences of the intervention, including soft tissue injuries and a broken ankle. Twenty-two studies (81%) reported on intention-to-treat analysis [28–32, 35, 36, 38–43, 45, 47–51, 53–55] or if they only included participants that were present at follow-up. Attrition was provided or could be calculated for all studies except one [34]. The median attrition rate (measured directly after the intervention) for the intervention group (range, 0%—33%) and the control group (range, 0%—45%) was 11%. Only two studies (7%) did not provide reasons for why participants dropped out of the intervention [31, 44].

### Adoption

All studies described the intervention location. For three studies, the intervention either started with centre-based training sessions [33, 55] or information sessions [46]. Three studies were delivered in a hybrid format, i.e., starting on-site and continuing at home [37–39]. Other than interaction with on-site staff during the centre-based component or introduction of the study and when outcomes were assessed, almost half (*n* = 11; 46%) of the home-based interventions were fully delivered via online apps and did not require the involvement of health professionals unless specifically requested by a participant [31–34, 40–45]. In the remaining 13 (54%) studies, researchers and health professionals, including nurses, physicians, physiotherapists, dieticians, and exercise specialists, were involved to review data, provide feedback and provide motivational reinforcement. With the exception of one study [47], any further characteristics of staff delivering the intervention were not reported.

### Implementation

Implementation was, together with maintenance, the least addressed dimension. While almost all studies reported on intervention duration and intervention frequency, none of the studies mentioned the ongoing cost of intervention implementation. Information on intervention fidelity was also scarce and reported in only three publications: Widmer et al. [37] reported that no changes were made after the initiation of the study; Pakrad et al. [46] mentioned some changes were made after protocol registration, without reporting detail; and Pfaeffli-Dale et al. [43] stated that the study protocol was amended to include an additional end point. Nine studies (33%) reported on the application of theories, models, or frameworks to guide the implementation and delivery of the intervention. Four (15%) [33, 42, 43, 46] used the social cognitive theory, a common behaviour-change theory applied in managing chronic health conditions. Seven studies (26%) [33, 37, 41, 46, 50, 51, 55] used behaviour change techniques or models to guide the delivery of the behaviour change interventions.

### Maintenance

Only 10 (37%) studies measured primary or secondary outcomes post intervention. In four (15%) studies [37, 44, 50, 51], follow-up assessment(s) took place three to six months after the intervention ended. A further four (15%) studies measured outcomes one year [28–30] or two years [39] after intervention completion. One study [55] and its additional publication [56] reported on outcome measures up to four years after the start of the intervention. Two (7%) studies reported on program maintenance. This included the sustainability of adaptations made following intervention completion [51] and continued use and monitoring of the e-health tool after six months [44]. None of the studies reported program maintenance costs or whether the program was adopted in another setting.

## Discussion

The aim of this review was to evaluate the implementation of digital-supported interventions for the secondary prevention of heart disease using the RE-AIM framework. We found that there were significant gaps in the reporting of internal and external validity factors at both the individual and organisational level. It may be that data related to specific RE-AIM dimensions were not reported because authors did not find these data relevant to the aim of the publication or there was an intent to report this in subsequent publications. Since the included studies were all clinical trials, data contributing to reach, effectiveness, and adoption should be reported, with implementation and maintenance more likely to be underreported.

### RE-AIM dimensions

Characteristics required to describe the Reach dimension, which is an individual level measure of participation, are not well reported. None of the 27 studies explicitly described the target population, a finding echoed in similar reviews [16, 17, 19, 57]. The use of convenience sampling methods in trials often makes it not possible to draw conclusions on the representativeness of the sample and consequently, limits generalisability of digital-supported interventions to different socio-demographic groups. The exclusion criteria of the studies, as defined elsewhere [13] placed several restrictions that reduced access for vulnerable populations, including the elderly, the culturally and linguistically diverse, and those with no access to a smart device or no connectivity to the Internet. Whitelaw et al. [58] also reported some common participant-level barriers in the uptake of digital health technologies in cardiovascular care, including difficult-to-use technology, poor Internet connection, and fear of using technology. The relatively low median participation rate (41%) among the studies implies the existence of participant-level access barriers. This rate is lower than the median or average rates provided by other systematic reviews reporting on the RE-AIM dimensions of digital health interventions, which range from 51%-70% [16, 17, 19]. Interestingly, the observed low participation rate is similar to traditional, face-to-face programs (REF). Digital-supported programs aim to increase access and uptake to secondary prevention care. This finding suggests that these novel programs may not be achieving their desired goals and more research is needed to understand the factors impacting participation. Further, attrition rates were low compared to traditional programs, suggesting that digital-supported programs may enhance adherence once patients participate. This may be an important factor explaining the benefits of mHealth supported secondary prevention programs and further supports the findings from our previous review [13].

All the studies evaluated were digital-supported interventions for secondary prevention of heart disease and consequently the primary and secondary outcome measures of the interventions were predominantly clinical and behavioural in nature. Reporting of broader outcomes, such as quality of life and participant satisfaction, was rare, even though these outcomes can provide insight into improving the quality of the delivered care as well as intervention compliance [59]. Critical evaluation of the impact of intervention delivery was less clear. In most of the studies, attrition was low, however, a few studies reported much higher attrition. Common reasons provided for attrition related to either clinical deterioration (being medically unwell or death) or participant withdrawal. Yet, it was not always apparent if a withdrawal was related to the use of the technology in the intervention or to other circumstances.

Improving the functional capacity, wellbeing, and health-related quality of life of people diagnosed with heart disease requires long-term lifestyle modifications, including adherence to prescribed medical treatments. However, any long-lasting effect on outcomes could not be assessed for most studies, as the long-term maintenance of individuals’ behaviour and health states was only reported in a minority of the studies. The ability of digital technology to improve care and increase uptake and equitable access to secondary prevention requires more evidence about long-term maintenance of effects.

Adoption assesses the setting and staff delivering the program. Reporting mainly focused on the intervention location and few details were provided about the characteristics of staff delivering the intervention, their willingness and motivation to be involved, and if any training was provided. Digital-supported interventions for the secondary prevention often include some clinical oversight and so a failure to report details of delivery agents fails to inform what resources may be needed to deliver future interventions. This makes translation difficult to other settings and as such, decreases the probability that the intended behaviour change will be adopted and maintained [20]. Typically, studies that examine clinical or behavioural outcomes fail to report the organisational level dimensions of the RE-AIM framework (adoption, implementation, and maintenance) [60]. To ensure that healthcare organisations enable their workforces to use digital methods in service delivery design and implementation must attempt to close the gap between an organizations’ adoption of digital methods relative to their digital ability [61].

Factors related to the implementation dimension provide insight into the feasibility and replicability of the intervention [60]. In our review, we found no available information on adaptations made prior to, during, and after program implementation, even though Stirman et al. [24] highlighted that this information is required to understand how to adapt interventions to different contexts while retaining critical components. In addition, almost no use was made of frameworks to inform the delivery of the interventions, while their use has been recognised as being vital to the implementation and sustainability of novel eHealth solutions, in which people, technology, and context are intertwined [62]. Similar to other reviews [17, 19, 20], none of the studies reported the costs of intervention implementation. This lack of reporting eliminates a monetary reference point for future researchers when considering designing similar strategies.

Overall, Maintenance was the most underreported dimension of the framework. Few studies reported maintenance of effect at the individual level. No study reported organisational level maintenance, which is the extent to which the intervention has become a part of routine health care practices [63]. Also, none of the included studies reported ongoing costs of program delivery. Teams designing interventions find planning for and assessing maintenance difficult [14], however, for an intervention to become institutionalised or part of the routine organisational practices, a greater emphasis on recognising external validity factors is needed. There may also be shortfalls in funding that cut interventions short and inhibit the evaluation of maintenance. The general lack of long-running large digital health projects hinder alternative insight into the durability of used digital resources [64] and therefore, the sustainability and generalisability of interventions.

The lack of information on external validity factors in most trials inhibits the evaluation of factors that influence program access and uptake and sustained implementation of interventions and their wider replication in different settings or with more diverse populations. Failing to report factors inhibits the opportunity to improve program uptake and access for disadvantaged populations. Additionally, none of the studies reported the costs involved with intervention implementation or the ongoing costs of program delivery, which eliminates the use of monetary information necessary for decision making. We recommend the use of hybrid trial designs that combine clinical effectiveness trials with implementation and have the potential to rapidly translate the evidence into real-world settings [65], and that to improve long-term sustainability and determine ongoing costs, intervention studies must report across all RE-AIM indicators.

### Improvements to the RE-AIM framework

Currently, there is a lack of consistency and accuracy in the reporting of many RE-AIM dimension indicators, hindering intervention replication and translation [23, 66]. In conducting our review, some modifications, including the addition of indicators, were made to the RE-AIM framework (described in the “Methods” section). The nature of digital-supported interventions necessitated these changes so that the framework could be applied to the specific characteristics, delivery, and implementation of such interventions. For example, additional information was collected about individuals delivering the intervention, such as the staff acceptability of the intervention. This addition was needed because the rapid development of digital health technologies is profoundly changing the way healthcare is delivered. Positive consumer acceptance and adoption (from both patients and clinicians) is impacting staff approaches to health occupations, tasks, and functions.

### Strengths and limitations

This review is the first that we are aware of to evaluate the process and implementation of digital-supported interventions in the secondary prevention of heart disease. By using a framework adapted to fit the specific characteristics of digital-supported interventions, we were able to systematically assess the extent to which each of the included studies reported on internal and external validity criteria. This process provided a comprehensive insight into the generalisability, translatability and scale-up of the interventions to wider populations and settings.

The changes made to the RE-AIM framework allows its application to the reviewed interventions, however, those changes have yet to be tested for reliability and validity.

While we attempted to systematically identify any publications that would provide additional RE-AIM data, it is possible that some were missed. Furthermore, we based our conclusions solely on the extent to which the RE-AIM indicators were reported in the publications and we did not contact authors for missing information. Finally, as we only included English peer-reviewed publications, we might have missed studies published in other languages.

## Conclusions

The lack of reporting of external validity factors in mHealth-supported interventions inhibits the evaluation of factors that influence program access and uptake and sustained implementation of interventions. As such, it cannot be assumed that reported outcomes are generalisable. A failure to report inhibits the opportunity to improve program uptake and access for disadvantaged populations and the wider replication in different settings or with more diverse populations. Additionally, none of the studies reported the costs involved with intervention implementation or the ongoing costs of program delivery, negating the use of financial information in decision making. We recommend that future approaches to digital supported secondary prevention of heart disease should adopt hybrid trial designs to report relevant RE-AIM dimensions and associated indicators to improve the translatability of empirical evidence to real-world settings.

### Supplementary Information

**Additional file 1: Supplementary Table 1.** Characteristics and RE-AIM reporting rates of reviewed studies.

## Data Availability

The datasets used and/or analysed during the current study are available from the corresponding author on reasonable request.

## Abbreviations

CR: Cardiac Rehabilitation
RE-AIM: Reach, Effectiveness, Adoption, Implementation, Maintenance
