# Supplementary material for: Optimising the implementation of digital-supported interventions for the secondary prevention of heart disease: a systematic review using the RE-AIM planning and evaluation framework
Source: BMC Health Serv Res. 2023 Dec 4;23:1347. doi: 10.1186/s12913-023-10361-6 (PMC10694952; doi:10.1186/s12913-023-10361-6)
Supplement: Supplementary file 1 — Additional file 1: Supplementary Table 1. Characteristics and RE-AIM reporting rates of reviewed studies. [file 12913_2023_10361_MOESM1_ESM.docx]

**Supplementary Table 1** Characteristics and RE-AIM reporting rates of reviewed studies

| **Study characteristics** | | | | | | | | **RE-AIM reporting rates (%) for the items in each dimension ^a^** | | | | | |
| --- | --- | --- | --- | --- | --- | --- | --- | --- | --- | --- | --- | --- | --- |
| **Reference** | **Country** | **Study design** | **Sample size** | **Study aim** | **Digital technology** | **Intervention setting** | **Staff involved** | **R (%)**  **(n=6)** | **E (%)**  **(n=8)** | **A (%)**  **(n=3)** | **I (%)**  **(n=4)** | **M (%)**  **(n=4)** | **Total (%)**  **(max. n=25)** |
| Bae, 2021^43^ | Korea | RCT | 879 | To modify the lifestyle and cardiovascular disease risk factors | SMS; Web application | Home | No | 83 | 75 | 100^e^ | 25 | 0 | 60 |
| Blasco, 2012^57^ ^b^ | Spain | RCT | 203 | To monitor several risk factors | SMS; Web application | Home | Yes: cardiologist | 67 | 88 | 67 | 25 | 0 | 56 |
| Chow, 2015^44^ | Australia | RCT | 710 | To examine the effect of a lifestyle-focused semi-personalized support program on risk factor modification | SMS; Web application | Home | No: cardiologist only on demand | 83 | 88 | 67 | 50 | 0 | 64 |
| Islam, 2019^70^ ^c^ |  |  |  | To examine the effect of a lifestyle-focused semi-personalized support program on depression scores |  |  |  |  |  |  |  |  |  |
| Frederix, 2015^41^ | Belgium | RCT | 80 | To evaluate the medium-term clinical effectiveness of a physical activity telemonitoring program | SMS; Email; Wearable device | Hybrid | Yes: during centre-based component | 67 | 63 | 100^e^ | 25 | 0 | 52 |
| Frederix, 2017^42^ | Belgium | RCT | 126 | To assess whether follow-up transitional telerehabilitation could induce long-term health benefits and remain cost efficient | SMS; Email; Web application; Wearable device | Hybrid | Yes: during centre-based component | 67 | 88 | 67 | 25 | 25 | 60 |
| Frederix, 2016^71^ ^c^ |  |  |  | To evaluate the cost effectiveness of a CR programme. |  |  |  |  |  |  |  |  |  |
| Khonsari, 2015^56^ | Malaysia | RCT | 62 | To evaluate the medium-term clinical effectiveness of a physical activity telemonitoring program on top of and after conventional CR. | Automated SMS; Telephone calls | Home | Yes: researcher follow-up telephone calls | 67 | 75 | 100^e^ | 25 | 0 | 56 |
| Khonsari, 2020^45^ | Iran | Fesibility RCT | 78 | To evaluate a mHealth intervention to increase medication adherence among Iranian coronary heart disease patients. | Automated SMS | Home | No | 67 | 88 | 100^e^ | 50 | 0 | 64 |
| Lear, 2014^32^ ^b^ | Canada | RCT | 78 | To test the clinical effectiveness of a virtual CRP (vCRP) delivered exclusively using Internet-based technology (short-term results) | Teleconference; Web application; Wearable device | Home | Yes: nurse, physical therapist/exercise specialist, dietician | 50 | 88 | 67 | 25 | 25 | 56 |
| Lear, 2015^72^ ^c^ |  |  |  | To compare a four-month vCRP with a 12-month sustainability follow-up on exercise capacity and risk factor reduction compared to usual care in patients living in small urban and rural communities without access to standard CR |  |  |  |  |  |  |  |  |  |
| Kraal, 2017^58^ | Netherlands | RCT | 90 | To improve exercise behaviour by providing feedback on exercise data using motivational interviewing principles. | Web application; Telephone calls | Home^d^ | Yes: physical therapist/exercise specialist | 67 | 88 | 67 | 50 | 25 | 64 |
| Kraal, 2014^73^ ^c^ |  |  |  | To evaluate home-based CR with telemonitoring guidance using coaching interventions including strategies for behavioural changes with the aim to maintain adherence to a healthy lifestyle and to improve long-term effects. | Telephone calls |  |  |  |  |  |  |  |  |
| Brouwers, 2022^60^ ^c^ |  |  |  | To evaluate the long-term effectiveness of the cardiac-telerehab intervention on physical activity levels, physical fitness and quality of life after 4 years of follow-up, as compared with centre-based CR after 4 yrs. | Web application |  |  |  |  |  |  |  |  |
| Maddison, 2019^59^ | New Zealand | RCT | 162 | To test the effectiveness and costs of real-time remotely monitored exercise-based cardiac telerehabilitation with traditional centre-based programmes among adults with CHD. | Mobile app; Web application; Telemonitoring; Wearable device | Home | Yes: physical therapist/exercise specialist | 67 | 88 | 67 | 50 | 25 | 64 |
| Marvel, 2021^36^ ^b^ | USA | RCT with historical control | 1064 | To determine if patients with acute myocardial infarction who use a guideline-based self-management digital health intervention have lower rates of all-cause 30-day readmissions compared with a historical control group | Mobile app; Wearable device | Home^d^ | No | 83 | 50 | 100^e^ | 50 | 0 | 56 |
| Shan, 2020^74^ ^c^ |  |  |  | To examine feasibility and the (1) proportion of post-AMI patients with controlled BP and hypotension, and (2) association between prior cardiovascular disease (CVD) and BP post-AMI. | Mobile app |  |  |  |  |  |  |  |  |
| Bhardwaj, 2021^75^ ^c^ |  |  |  | To conduct an economic evaluation, from the hospital perspective, of the cost effectiveness of the Corrie digital health intervention plus standard of care in reducing 30-day readmissions when compared with standard of care alone in acute myocardial infarction care. | Mobile app |  |  |  |  |  |  |  |  |
| McElroy, 2016^37^ | USA | Prospective cohort study | 443 | To determine the added benefit of incorporating digital health kit into a formal readmissions reduction program in preventing 30-d readmissions and to evaluate patient and provider satisfaction with the use of these devices. | Web application; Wearable device | Home | No: healthcare provider only on demand | 33 | 50 | 67 | 25 | 0 | 36 |
| Pakrad, 2021^49^ | Iran | RCT | 81 | To compare traditional (1-month supervised) vs hybrid cardiac rehabilitation (CR; usual care) with an additional 3 months offered | Mobile app | Home^d^ | Yes: physical therapist/exercise specialist | 67 | 63 | 67 | 63 | 0 | 54^f^ |
| Pfaeffli, 2015^46^ | New Zealand | RCT | 123 | To investigate the effectiveness of a mHealth-delivered comprehensive CR program to improve adherence to recommended lifestyle behaviors in addition to traditional CR | SMS; Web application; Wearable device | Home | No | 83 | 75 | 100^e^ | 75 | 0 | 68 |
| Reid, 2012^33^ | Canada | RCT | 223 | To investigate whether patients who used the CardioFit programme were more physically active 6- and 12-months following hospitalization for CHD than patients who received physical activity advice from their cardiologist. | Email; Web application | Home | Yes: physical therapist/exercise specialist | 67 | 75 | 67 | 25 | 25 | 56 |
| Riegel, 2020^38^ | USA | RCT | 130 | To compare the efficacy of a mobile application rooted in behavioural economics on medication adherence. | Mobile app; SMS | Home | No | 50 | 63 | 100^e^ | 25 | 0 | 48 |
| Sherrard, 2009^34^ ^b^ | Canada | RCT | 331 | To determine if interactive voice response can improve medication compliance and reduce adverse events as patients transition from hospital to home among postoperative cardiac surgical patients. | Automated telephone calls | Home | No: nurse only on demand | 83 | 75 | 67 | 25 | 0 | 56 |
| Sherrard, 2015^35^ ^b^ | Canada | RCT | 1608 | To evaluate whether the use of an interactive voice response follow-up system improved ACS best practice guideline compliance. | Automated telephone calls | Home | No: nurse only on when required | 83 | 75 | 67 | 25 | 0 | 56 |
| Snoek, 2019^50^ | Netherlands | RCT | 122 | To assess the acute and sustained effects of a six-month heart-rate-based telerehabilitation programme, following the completion of CR in patients with coronary artery disease | Mobile app; Telephone calls; Wearable device; | Home | Yes: nurse | 83 | 75 | 67 | 25 | 25 | 60 |
| Snoek, 2021^54^ ^b^ | Netherlands | RCT | 179 | To assess whether a 6-month guided mobile CR program is an effective therapy for patients 65 years or older who decline participation in a conventional CR program. | Telephone calls; Wearable device | Home | Yes: not specified | 83 | 88 | 67 | 50 | 50 | 72 |
| Treskes, 2020^51^ ^b^ | Netherlands | RCT | 200 | To investigate whether smart technology in clinical practice can improve BP regulation and to evaluate the feasibility of such an intervention. | Mobile app; Videoconference | Home | Yes: nurse, physician | 67 | 88 | 67 | 25 | 0 | 56 |
| Treskes, 2022^76^ ^c^ |  |  |  | To describe a cost-utility analysis of the eHealth intervention compared to regular follow-up in the outpatient care setting of patients who have been treated for AMI with primary percutaneous |  |  |  |  |  |  |  |  |  |
| Volpp, 2017^39^ ^b^ | USA | RCT | 1503 | To determine whether a system of medication reminders using financial incentives and social support delays subsequent vascular events in patients following AMI compared with usual care. | Web application; Electronically monitored pill bottles | Home | Yes: staff engagement advisor | 83 | 63 | 67 | 13 | 0 | 50^f^ |
| Widmer, 2017^40^ | USA | RCT | 71 | To determine whether digital health intervention administered during CR would reduce CV-related emergency department visits and rehospitalizations in patients after percutaneous coronary intervention for acute coronary syndrome. | Mobile app; Web application | Hybrid | Yes: for centre-based component | 50 | 75 | 67 | 75 | 25 | 60 |
| Widmer, 2020^77^ ^c^ |  |  |  | To evaluate digital health intervention log-in patterns |  |  |  |  |  |  |  |  |  |
| Wolf, 2016^47^ | Sweden | Substudy of RCT | 142 | To investigate the effect of an eHealth diary and symptom-tracking tool in combination with person centred care for patients with acute coronary syndrome. | Mobile app; Web application | Home | No | 67 | 38 | 100^e^ | 25 | 50 | 52 |
| Woodend, 2008 ^31^ | Canada | RCT | 128 | To determine whether tele-home monitoring of patients with cardiac disease at high risk of readmission would reduce hospital re-admissions, improve functional status, and improve quality of life over usual care | Videoconference via telephone line | Home | Yes: Nurse | 33 | 88 | 67 | 25 | 25 | 52 |
| Yu, 2020 ^52^ ^b^ | China | RCT | 1000 | To evaluate the effectiveness and feasibility of using a smartphone-based application to improve medication adherence in patients after coronary artery bypass graft. | Mobile app | Home | Yes: nurse | 50 | 75 | 67 | 25 | 0 | 48 |
| Yudi, 2021^48^ | Australia | RCT | 168 | To assess whether a smartphone-based, early CR program improved exercise capacity in patients with ACS. | Mobile app | Home | No | 67 | 75 | 100^e^ | 25 | 0 | 56 |
|  |  |  |  |  | **Average reporting rates across the studies** | | | **67** | **75** | **82** | **36** | **11** | **57** |

^a^ R: reach; E: efficacy/effectiveness; A: adoption; I: implementation; M: maintenance

^b^ This study was excluded in the previous systematic review

^c^ Companion paper

^d^ Home-based mHealth intervention with a few centre-based training or information sessions at the start of the intervention

^e^ This study was fully automated, i.e., no staff was involved in the delivery of the intervention. They were rated out of n=2 items.

^f^  Half marked if fidelity was mentioned without any further details, or if intervention duration was provided without further details
